# Rapid yield optimization of miniaturized microwave passives by response features and variable-fidelity EM simulations

**DOI:** 10.1038/s41598-022-26562-8

**Published:** 2022-12-23

**Authors:** Anna Pietrenko-Dabrowska, Slawomir Koziel

**Affiliations:** 1 Faculty of Electronics, Telecommunications and Informatics, Gdansk University of Technology, 80-233 Gdańsk, Poland; 2 Engineering Optimization and Modeling Center, Reykjavik University, 102 Reykjavík, Iceland

**Keywords:** Electrical and electronic engineering, Computational science

## Abstract

The operation of high-frequency devices, including microwave passive components, can be impaired by fabrication tolerances but also incomplete knowledge concerning operating conditions (temperature, input power levels) and material parameters (e.g., substrate permittivity). Although the accuracy of manufacturing processes is always limited, the effects of parameter deviations can be accounted for in advance at the design phase through optimization of suitably selected statistical performance figures. Perhaps the most popular one is the yield, which provides a straightforward assessment of the likelihood of fulfilling performance conditions imposed upon the system given the assumed deviations of designable parameters. The latter are typically quantified by means of probability distributions pertinent to the fabrication process. The fundamental obstacle of the yield-driven design is its high computational cost. The primary mitigation approach nowadays is the employment of surrogate modeling methods. Yet, a construction of reliable metamodels becomes problematic for systems featuring a large number of degrees of freedom. Our work proposes a technique for fast yield optimization of microwave passives, which relies on response feature technology as well as variable-fidelity simulation models. Utilization of response features enables efficient handling of issues related to the system response nonlinearities. Meanwhile, the incorporation of variable-resolution simulations allows for accelerating the yield estimation process, which translates into remarkably low overall cost of the optimizing the yield. Our approach is verified with the use of three microstrip couplers. Comprehensive benchmarking demonstrates its superiority in terms of computational efficiency over the state-of-the-art algorithms, whereas reliability is corroborated by electromagnetic-driven Monte Carlo simulations.

## Introduction

Standard microwave design procedures, including parametric optimization, normally neglect possible deviations of dimensions and material parameters from the nominal values thereof. At the same time, manufacturing processes are always imperfect, and so is the knowledge pertaining to the material properties (e.g., substrate relative permittivity) or operational conditions (possible geometrical distortions of the device, temperature, signal power level, etc.). All of them may exert some, typically undesirable, effects on electrical characteristics of the device^1^. The uncertainties pertinent to operating conditions (also referred to as epistemic^2^) are systematic and can be accounted for by ensuring satisfactory system performance within the prescribed condition range (e.g., input signal level). On the other hand, fabrication tolerances, e.g., inaccuracy of chemical etching for PCB-based components, or mechanical milling for waveguide structures, are of stochastic nature. These have to be described by means of process-specific probability distributions, typically Gaussian of a given mean and covariance matrix. As the performance requirements imposed on contemporary microwave devices are often stringent, tolerance-induced deviations of designable parameters from their nominal values may readily result in the system failing to meet the specifications. Consequently, the ability to quantify the effects of uncertainties is instrumental in estimating the design robustness, and, even more importantly, robustness enhancement. The necessitates optimization of suitably formulated statistical performance figures^3^, and is often labeled as tolerance-aware design^4^, robust design^5^, or design centering^6^. For microwave circuits, the performance requirements are typically formulated in a minimax form (e.g., through acceptance thresholds for return loss, transmission, power split, or phase responses), which makes yield^7^ one of the most appropriate metrics of design robustness.

Evaluation of the system robustness under fabrication tolerances requires statistical analysis^8^, which is a CPU-intensive procedure when conducted utilizing full-wave electromagnetic (EM) simulations. At the same time, EM analysis is required to maintain reliability. The latter may be especially critical for compact microwave passives that normally feature considerable cross-coupling effects being byproducts of such miniaturization strategies as transmission line meandering^9^ or exploiting the slow-wave phenomenon^10^. Excessive computational cost of direct EM-driven statistical analysis (e.g., Monte Carlo simulation) fostered the development of accelerated methods. One possibility is to reduce the problem complexity (e.g., worst case analysis^11^), yet accuracy degradation may be unacceptable. As of now, the most popular uncertainty quantification methods rely on surrogate modeling techniques, which enable massive system evaluations at minimum expenses. Some of widely used techniques include polynomial chaos expansion (PCE)^12,13^, neural networks^14^, and response surface approximation^15^. Unfortunately, utilization of data-driven surrogates is encumbered by the curse of dimensionality^16^, which may be mitigated to a certain extent by dimensionality reduction through, e.g., the employment of principal component analysis^17^), improved modeling schemes (e.g., PC kriging^18^), usage of multi-fidelity simulations (e.g., co-kriging^19^), or the usage of physics-based surrogates such as space mapping^20^.

The quantification of the effects of uncertainties is a foundation of robust design procedures (yield-driven optimization^21^, tolerance-aware design^22^), which aim at improving the system immunity to manufacturing and other tolerances. In particular, optimization of yield attempts to directly increase the likelihood of fulfilling the performance conditions imposed upon the circuit under the assumed parameter deviations. Robust optimization can also be considered in a geometrical sense (by placing the design near the center of the feasible region), or addressed by increasing the acceptable levels of tolerances that ensure meeting the specifications (e.g., tolerance hypervolume maximization^23^). Again, the biggest challenge of EM-driven statistical design are computational expenditures entailed by repetitive circuit simulations. Most practical optimization frameworks employ surrogate modeling methods^24–32^ both data-driven^24,25^, and physics-based^30^. In some cases, when the considered statistical figures of merit are functions of statistical moments of the response of the system at hand, polynomial chaos expansion models can be used to avoid numerical integration of the underlying probability distributions^33^. For microwave components and minimax-type of specifications, Monte Carlo analysis^34^ carried out with the aid of the surrogate may be unavoidable to evaluate the robust objective function. Rendering reliable metamodels for yield optimization is, however, more demanding than for statistical analysis purposes due to a larger part of the parameter space that has to be covered. The alleviation is possible by adopting iterative approaches (e.g., sequential approximate optimization^35^), in which the model is locally rendered in the proximity to the current design, thereby the training data acquisition cost may be kept low. Meanwhile, the model domain is relocated accordingly in the course of the optimization procedure. The response feature technology^36^ offers alternative way of handling the problem, in which the design specifications are formulated at the tier of suitably defined characteristic (feature) points of the circuit outputs. Close-to-linear relationship between the feature points coordinates and circuit dimensions facilitates a surrogate model construction, thereby enabling acceleration of various design procedures^37^.

This paper introduces a technique for fast yield optimization of microwave passive components, which capitalizes on the feature-based framework reported in^38^, and further expedites a robust design process by incorporating variable-resolution EM models. Following^38^, our methodology utilizes local feature-based surrogates, employed for cost-effective estimation of the circuit yield, and embeds the search process in the trust region framework to control design relocation, and to ensure convergence. An additional speedup is achieved by estimating response feature sensitivity at the level of low-resolution EM analysis. Due to reasonably good correlations between EM models of various fidelities, zero-order correction turns sufficient to ensure acceptable surrogate accuracy. As a result, the computational expenses of the robust design procedure is cut down, and amounts to a handful of EM high-fidelity simulations of the circuit at hand. Comprehensive verification experiments carried out for three microstrip couplers demonstrate superior performance of the proposed method over the benchmark. The latter include several recent surrogate-assisted techniques, as well as the feature-based approach of^38^.

## Yield optimization of microwave passives using multi-fidelity response features

This section delineates the proposed yield optimization algorithm. Background information concerning nominal (“Nominal optimization problem formulation” section) and robust design problem formulation (“Yield optimization” section) is followed by recalling the concept and the properties of response features (“Response features for low-cost yield estimation” section), as well as variable-resolution computational models (“Variable-resolution EM models for further cost reduction” section). The variable-fidelity feature-based surrogates are discussed in “Yield optimization using variable-resolution feature-based surrogates” section, whereas the complete optimization procedure is summarized in “Complete algorithm” section.

### Nominal optimization problem formulation

The fabrication yield is defined with regard to design specifications pertinent to the microwave component of interest. These, in turn, are defined using a set of conditions for the scattering parameters (or the functions thereof). In this paper, the verification structures considered in “Numerical verification” section are branch-line and rat-race couplers, therefore, the specific performance requirements used to illustrate the discussed concept will pertain to this type of circuits. For couplers, the effects of manufacturing inaccuracies manifest themselves as modifications of the power split ratios, but also relocation of the operating frequencies/bandwidths. Maximization of yield aims at adjusting the system geometry parameters to enhance the probability of fulfilling the specs given the parameter deviations. All of these will be rigorously formulated in the remaining part of this section.

Consider an *N*-band coupler described by *n* geometry parameters aggregated into a vector ***x*** = [*x*_1_ … *x*_*n*_]^*T*^. Further, let [*f*_*L.k*_* f*_*R.k*_] denote the *k*th operating band, *k* = 1, …, *N*, with *f*_*L.k*_ and *f*_*R.k*_ being the lower and upper ends of the band, respectively. Within that band, the matching and isolation characteristics are not to exceed a user-defined value *S*_max_ (e.g., − 20 dB).

Finally, let *D*_*k*_ be the tolerance thresholds for the power split deviations at the center frequency *f*_0*.k*_ = [*f*_*R.k*_ + *f*_*L.﻿k*_]/2, and *S*_*k*_ stand for the intended power split ratio. The following conditions are to be fulfilled for the design ***x*** to satisfy the aforementioned specifications, where *S*_*k*1_(***x***,*f*) stands for the respective *S*-parameter response, *k* = 1, …, 4:

1 $$\max \left\{ {f \in \bigcup\nolimits_{k = 1}^{N} {\left[ {f_{L.k} ,f_{R.k} } \right]} :|S_{11} ({\mathbf{x}},f)|} \right\} \le S_{\max }$$

2 $$\max \left\{ {f \in \bigcup\nolimits_{k = 1}^{N} {\left[ {f_{L.k} ,f_{R.k} } \right]} :|S_{41} ({\mathbf{x}},f)|} \right\} \le S_{\max }$$

3 $$\left| {|S_{31} ({\mathbf{x}},f_{0.k} )| - |S_{21} ({\mathbf{x}},f_{0.k} )| - S_{k} } \right| \le D_{k} \quad k = 1, \ldots ,N$$

The fulfillment of (1)–(3) is analogous to the circuit operating at the required bandwidth and ensuring assumed power division for all frequency intervals [*f*_*L.k*_* f*_*R.k*_].

Although the conditions (1)–(3) define the minimum requirements, it is normally possible, through optimization, to improve the circuit performance further. This can be done, e.g., by improving both matching and isolation over the target bandwidths, or to broaden the bandwidths at the *S*_max_ level. The design that is optimized in the former sense, will be referred to as the nominal one, and denoted as ***x***^(0)^. It is obtained by solving

4 $${\mathbf{x}}^{(0)} = \arg \mathop {\min }\limits_{{\mathbf{x}}} \left\{ {\max \left\{ {f \in \bigcup\nolimits_{k = 1}^{N} {\left[ {f_{L.k} ,f_{R.k} } \right]} :\max \{ |S_{11} ({\mathbf{x}},f)|,|S_{41} ({\mathbf{x}},f)|\} } \right\}} \right\}$$

subject to the equality constraint

5 $$|S_{31} ({\mathbf{x}},f_{0.k} )| - |S_{21} ({\mathbf{x}},f_{0.k} )| = S_{k} \quad k = 1, \ldots ,N$$

The design ***x***^(0)^ refers to the best achievable design that can be attained without considering manufacturing tolerances. In this work, it will be used as a starting point for yield optimization.

### Yield optimization

Let *d****x*** denote a vector of deviations of the circuit parameters from their nominal values. The deviations originate from manufacturing inaccuracy and are quantified using probability distributions that are specific to the fabrication process. In this work, we assume joint Gaussian distribution with zero mean and variance *σ* (common for all parameters). In a more generic setup, the distribution can be determined by a covariance matrix that accounts for the circuit topology (e.g., correlations between deviations for certain parameters).

The fabrication yield *Y* is computed by integrating the probability density function *p*(***x***,***x***^(0)^) that describes deviations of ***x*** from the nominal design ***x***^(0)^. The integration is executed over the set *X*_*f*_, which contains all designs satisfying the performance specifications (e.g., conditions (1) through (3) for the considered coupling structure). We have^39^

6 $$Y({\mathbf{x}}^{(0)} ) = \int\limits_{{X_{f} }} {p({\mathbf{x}},{\mathbf{x}}^{(0)} )d{\mathbf{x}}}$$

As the geometry of the feasible set is not known explicitly, in practice, the yield is estimated through numerical integration, most often by employing Monte Carlo simulation. Given a set of random observables *d****x***^(*k*)^, *k* = 1, …, *N*_*r*_, allocated according to the density function *p*, we get

7 $$Y({\mathbf{x}}^{(0)} ) = N_{r}^{ - 1} \sum\nolimits_{k = 1}^{{N_{r} }} {H({\mathbf{x}}^{(0)} + d{\mathbf{x}}^{(k)} )}$$

where *H*(***x***) = 1 if the performance specifications are satisfied for the design ***x***, *H*(***x***) = 0 if the specs are violated.

The yield optimization task is formulated as

8 $${\mathbf{x}}^{*} = \arg \mathop {\min }\limits_{{\mathbf{x}}} \{ - Y({\mathbf{x}})\}$$

Solving (8) entails repetitive yield estimations, which is associated with a prohibitive CPU cost when realized directly with the use of EM analysis. As elaborated on in the introduction, the majority of practical approaches employ fast surrogate models for the purpose of evaluating (7). However, building the surrogate can also generate considerable expenses and be numerically challenging for circuits described by many parameters. This paper incorporates two mechanisms intended to mitigate these issues: a response feature approach (“Response features for low-cost yield estimation” section) and variable-resolution EM models (“Variable-resolution EM models for further cost reduction” section).

### Response features for low-cost yield estimation

Following^38^, the yield estimation while solving the problem (8) is expedited using a response feature technology^36^. Feature-based modeling^40^ and optimization^41^ benefits from re-formulating the design problem in terms of properly selected characteristic points of the circuit outputs, and weakly nonlinear dependence between the frequency and level coordinates of these points on circuit dimensions. This section discusses how the performance specifications considered in “Nominal optimization problem formulation” section can be expressed in terms of response features, which will be used in “Yield optimization using variable-resolution feature-based surrogates” section to realize reduced-cost yield maximization.

**Figure 1:**
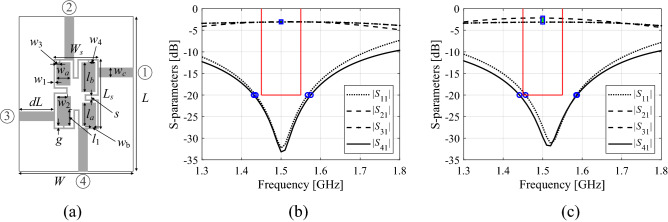
Conceptual illustration of response features for a microwave coupler: *S*-parameters and feature points representing − 20 dB level of |*S*_11_| (matching) and |*S*_41_| (isolation) responses (o), along with |*S*_21_| and |*S*_31_| (transmission) at the intended operating frequency *f*_0_ = 1.5 GHz (verbal description: square). The characteristic point coordinates permit assessment of performance requirements satisfaction (100 MHz matching/isolation bandwidth centered around *f*_0_), and maximal power split deviation of 0.5 dB at *f*_0_): (**a**) coupler geometry, (**b**) design fulfilling specs, (**c**) design infringing power split and bandwidth conditions.

The choice of the feature points depends on design specifications assumed for the system. Going back to the coupler example of “Nominal optimization problem formulation” section (cf. conditions (1)–(3)), it can be concluded that the feature points should account for − 20 dB levels for the matching and isolation responses, and also for the power split at the center frequency. Figure 1 illustrates this case for an exemplary branch-line coupler. Note that for certain feature points the relevant information is carried by their frequency coordinates, level coordinates, or both.

Throughout this paper, the feature points data will form a feature vector ***P***, the entries of which will be the frequency and/or level coordinates of corresponding features. For the considered coupler example, we have

9 $${\mathbf{P}}({\mathbf{x}}) = [p_{1} ({\mathbf{x}})p_{2} ({\mathbf{x}}) \ldots p_{6} ({\mathbf{x}})]^{T} = [f_{1} ({\mathbf{x}})f_{2} ({\mathbf{x}})f_{3} ({\mathbf{x}})f_{4} ({\mathbf{x}})l_{1} ({\mathbf{x}})l_{2} ({\mathbf{x}})]^{T}$$

In (9), the frequencies *f*_1_ through *f*_4_ correspond to the − 20 dB level of |*S*_11_| (*f*_1_ and *f*_2_) and |*S*_41_| (*f*_3_ and *f*_4_), whereas *l*_1_ and *l*_2_ are the levels of the transmission characteristics |*S*_21_| and |*S*_31_|, respectively, at the (target) coupler center frequency. This can be generalized to the example of a multi-band coupler, where we have

10 $$\begin{aligned} & {\mathbf{P}}({\mathbf{x}}) = [p_{1} ({\mathbf{x}})p_{2} ({\mathbf{x}}) \ldots p_{6N} ({\mathbf{x}})]^{T} \\ & \quad = [f_{1.1} ({\mathbf{x}})f_{2.1} ({\mathbf{x}})f_{3.1} ({\mathbf{x}})f_{4.1} ({\mathbf{x}})l_{1.1} ({\mathbf{x}})l_{2.1} ({\mathbf{x}}) \ldots f_{1.N} ({\mathbf{x}})f_{2.N} ({\mathbf{x}})f_{3.N} ({\mathbf{x}})f_{4.N} ({\mathbf{x}})l_{1.N} ({\mathbf{x}})l_{2.N} ({\mathbf{x}})]^{T} \\ \end{aligned}$$

The secondary subscript is the index of the circuit band. In general, the composition of the feature vector is problem- and performance-specification-dependent. The coordinates of the feature vector are garnered from EM-simulated characteristics of the circuit at hand.

Having defined the vector ***P***, we are in a position to reformulate the performance requirements (conditions (1) through (3)) in terms of its coordinates:

11 $$f_{1.k} ({\mathbf{x}}) \le f_{L.k} ,\quad f_{3.k} ({\mathbf{x}}) \le f_{L.k} ,\quad k = {1}, \ldots ,N$$

12 $$f_{2.k} ({\mathbf{x}}) \ge f_{R.k} ,\quad f_{4.k} ({\mathbf{x}}) \ge f_{R.k} ,\quad k = { 1}, \ldots ,N$$

13 $$|l_{1.k} ({\mathbf{x}}) - l_{2.k} ({\mathbf{x}})| \le D_{k} ,\quad k = {1}, \ldots ,N$$

As mentioned at the beginning of this section, the benefit of replacing (1)–(3) by (11)–(13) is that the relationships between the feature point coordinates and circuit dimensions are to a smaller degree nonlinear than an analogous relationship for the entire frequency characteristics. This facilitates a construction of surrogate models but also leads to accelerating the optimization processes.

### Variable-resolution EM models for further cost reduction

**Figure 2:**
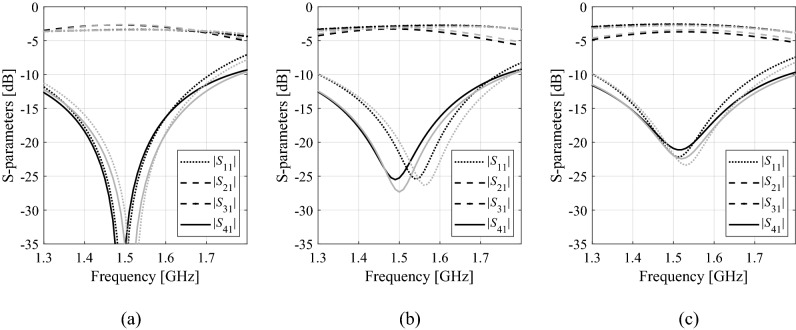
*S*-parameters for the coupler considered in Fig. 1 evaluated using the low- (gray) and high-fidelity (black) EM simulation. Observe considerable misalignment, especially in terms of the frequency shifts, yet the overall shape and the amount of misalignment is consistent for different designs shown in the pictures (plots (**a**) through (**c**)).

The second mechanism used in this work to accelerate the robust design process lies in the employment of multi-fidelity EM models. Reducing the discretization density of the structure allows for speeding up the process of simulation yet with detrimental effect to accuracy, which can manifest itself through the frequency and/or level shifts of the circuit characteristics. On the other hand, as the underlying physics of both low- and high-fidelity is the same, the EM models of different resolutions are normally well correlated, assuming that the model resolution is not pushed too far. This is illustrated in Fig. 2 for the coupler of Fig. 1a, where we can observe low- and high-fidelity model responses for three randomly generated designs. In this case, the high-fidelity model features about 80,000 mesh cells, whereas the low-fidelity model is set up with approximately 20,000 cells.

**Figure 3:**
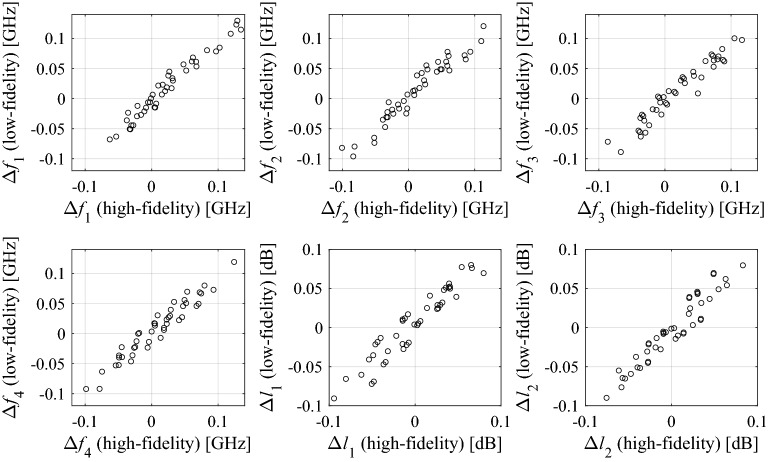
Scatter plots of feature coordinate differentials Δ*p*_*j*_ = *p*_*j*_(***x***_*k*_) − *p*_*j*_(***x***_*l*_) computed for all pairs of ten designs {***x***_*k*_}_*k* = 1,…,10_, generated in the vicinity of a nominal vector ***x***^(0)^ for a microwave coupler considered in Fig. 1. Horizontal and vertical axis correspond to the differentials of the high- and low-fidelity model, respectively.

As explained in “Response features for low-cost yield estimation” section, in this work, the robust design problem will be solved at the level of response features, therefore, we are mainly interested in correlation between the coordinates of the feature points of low- and high-fidelity models, or, more specifically, the feature point sensitivities. Figure 3 shows the differentials Δ*p*_*j*_ = *p*_*j*_(***x***_*k*_) − *p*_*j*_(***x***_*l*_) of the high- and low-fidelity model computed for all pairs of designs selected from a ten-element random set {***x***_*k*_}_*k* = 1, …, 10_, generated in the vicinity of a nominal vector ***x***^(0)^ for a coupler of Fig. 1a.

The features are selected as presented in Fig. 1 (see also (9)). It can be observed that the correlation between the models is excellent with the linear correlation coefficients equal to 0.99, 0.97, 0.97, 0.97, 0.96, and 0.96 for *f*_1_, *f*_2_, *f*_3_, *f*_4_, *l*_1_, and *l*_2_, respectively. This means that response feature sensitivity predicted by the low-fidelity model will be in a good agreement with sensitivity evaluated with the high-fidelity one. Still, the computational expenditures associated with sensitivity estimation is considerably lower, typically, by a factor three to four.

### Yield optimization using variable-resolution feature-based surrogates

The core of the proposed yield optimization follows the technique introduced in^38^, which is enhanced by utilization of the variable-resolution EM models. The yield is maximized in an iterative fashion using the trust-region (TR) procedure^42^.

In every iteration, a new design ***x***^(*i*+1)^ being an approximation of the optimum vector x^*^ is produced, *i* = 0, 1, … (here, the nominal design ***x***^(0)^ is the starting point), by solving

14 $${\mathbf{x}}^{(i + 1)} = \arg \mathop {\min }\limits_{{||{\mathbf{x}} - {\mathbf{x}}^{(i)} || \le d^{(i)} }} \{ - Y_{P} ({\mathbf{x}})\}$$

The yield *Y*_*P*_ is computed at the level or response features as in (11)–(13), with the feature point coordinates predicted using a linear (regression) model ***L***^(*i*)^ of ***P***(***x***) comprising the response features

15 $${\mathbf{L}}^{(i)} ({\mathbf{x}}) = {\mathbf{P}}({\mathbf{x}}^{(i)} ) + {\mathbf{J}}_{P.L} ({\mathbf{x}}^{(i)} ) \cdot ({\mathbf{x}} - {\mathbf{x}}^{(i)} )$$

in which ***J***_*P.L*_ is the approximation of the Jacobian matrix ***J***_*P*_

16 $${\mathbf{J}}_{P} ({\mathbf{x}}^{(i)} ) = \left[ {\frac{{\partial p_{j} ({\mathbf{x}}^{(i)} )}}{{\partial x_{k} }}} \right]_{\begin{subarray}{l} j = 1, \ldots ,2N \\ k = 1, \ldots ,n \end{subarray} }^{T}$$

estimated by finite differentiation with the use of the low-fidelity EM model. Let us denote the time evaluation ratio *M* between the high- and low-fidelity model. The cost of constructing the model ***L***^(*i*)^ equals to 1 + *n*/*M*, where *n* is the parameter space dimensionality. Assuming conservatively that *M* = 3, the computational savings reach sixty percent for *n* = 10.

Adjusting the size parameter *d*^(*i*)^ is an important consideration in TR frameworks. It is normally done based on the gain ratio *r*, which is defined as a ratio between the observed improvement of the merit function (here, the yield) and the improvement predicted by the linear model. It should be noted that evaluation of the yield at the candidate design ***x***^(*i*+1)^ requires rebuilding of the linear model at ***x***^(*i*+1)^, which would turn to be a waste of computational resources if *r* < 0, i.e., if the candidate design is rejected (according to the TR principles^42^, the design ***x***^(*i*+1)^ is only retained if *r* > 0). For the sake of cost savings, in this work, the gain factor is evaluated as *r* = [*Y*_*P#*_(***x***^(*j*+1)^) − *Y*_*P*_(***x***^(*j*)^)]/[*Y*_*P*_(***x***^(*j*+1)^) − *Y*_*P*_(***x***^(*j*)^)], where *Y*_*P#*_ is obtained from a linear model defined as in (15); however, instead of ***P***(***x***^(*i*)^) the vector ***P***(***x***^(*i*+1)^) derived from EM-evaluated system output for ***x***^(*i*+1)^. This only requires one EM analysis. The approximation is due to using the same Jacobian matrix for both ***x***^(*i*)^ and ***x***^(*i*+1)^, which is tenable as the distance between these two vectors is normally small (comparable to or smaller than the maximum assumed parameter deviation).

The actual yield estimation with the linear predictor ***L***^(*i*)^ is executed as Monte-Carlo-based integrating of (6), with the use of a large number of randomly allocated observables to reduce the estimation variance. For this purpose, the feature-based performance specifications (11)–(13) are verified for the output of the model ***L***^(*i*)^ obtained for each observable generated using the probability distribution assumed for input tolerances. The cost of this process is small in comparison to EM simulation of the circuit.

### Complete algorithm

**Figure 4:**
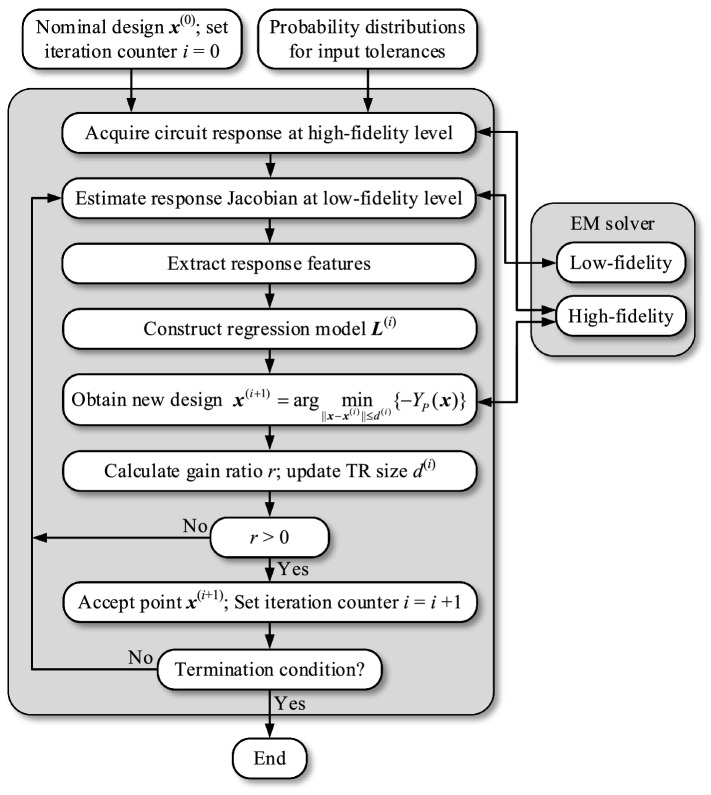
Flow diagram of the proposed yield optimization procedure.

The flow diagram of the yield enhancement procedure introduced in this section has been presented in Fig. 4. As emphasized before, we use the nominal design ***x***^(0)^ as the starting point for robust design process. The high-resolution EM model is only utilized to evaluate the circuit response at the current design, whereas response feature sensitivities are estimated using the low-fidelity model. The linear regression model is constructed upon extracting feature point coordinates, and utilized to obtain the candidate design ***x***^(*i*+1)^. The gain ratio is evaluated as explained in “Yield optimization using variable-resolution feature-based surrogates” section. It is used to decide about the acceptance of the candidate design and to adjust the search region size parameter *d*^(*i*)^. The algorithm is terminated if either ||***x***^(*i*+1)^ − ***x***^(*i*)^||< *ε* (convergence in argument), or *d*^(*i*+1)^ < *ε* (reduction of the TR size). In the numerical experiments of “Numerical verification” section, we employ the termination threshold *ε* = 10^−3^.

## Numerical verification

The proposed yield optimization framework is validated using three examples of microstrip couplers. It is also compared to four surrogate-assisted methods and the feature-based approach of^38^. The optimization procedure reliability is verified via Monte Carlo analysis performed with the use of the EM simulation models of the respective structures.

### Case studies

**Figure 5:**
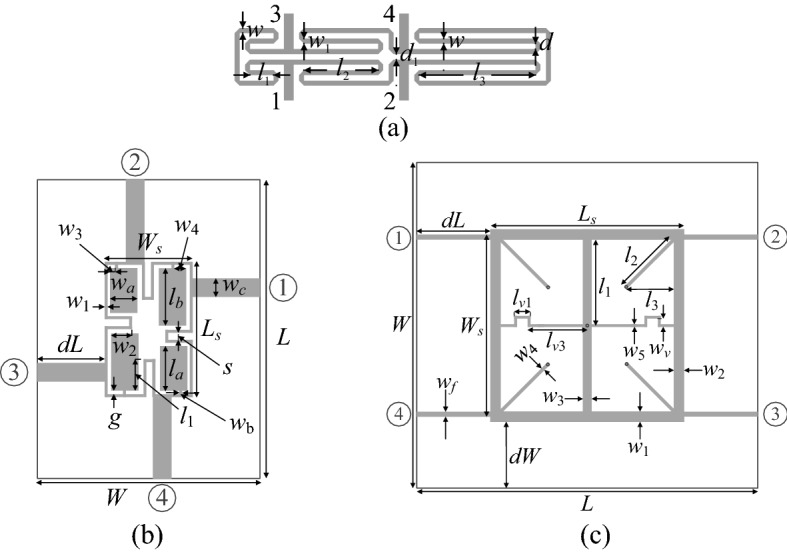
Verification circuits used for validation of the yield optimization framework of “Yield optimization of microwave passives using multi-fidelity response features” section: (**a**) miniaturized rat-race coupler with folded transmission lines (Coupler I)^43^, (**b**) compact branch-line coupler (Coupler II)^44^, (**c**) dual-band branch-line coupler (Coupler III)^45^.

In order to validate the yield optimization procedure presented in this work, we use three microstrip couplers, a miniaturized rat-race coupler (Coupler I)^43^ (Fig. 5a), a compact branch-line coupler, BLC (Coupler II)^44^ (Fig. 5b), along with a dual-band branch-line coupler (Coupler III)^45^ (Fig. 5c). Table 1 gathers the necessary data on all circuits, which include independent geometry parameters, dielectric substrates, the setup of low- and high-fidelity models, performance specifications, as well as nominal designs. In all cases, the computational models are evaluated using the time-domain solver of CST Microwave Studio. Also, in all cases, the input parameter tolerances are assumed to follow independent zero-mean Gaussian distributions with 0.017 mm variance, and maximum deviations limited to d_max_ = 0.05 mm.

**Table 1:** Validation circuits.

	Case study^$^		
	Circuit I	Circuit II	Circuit III
Substrate	RO4003 (*ε*_*r*_ = 3.38, *h* = 0.76 mm)	AD300 (*ε*_*r*_ = 2.97, *h* = 0.76 mm)	RO4003 (*ε*_*r*_ = 3.5, *h* = 0.51 mm)
Design parameters	***x*** = [*l*_1_ *l*_2_ *l*_3_ *d w w*_1_]^*T*^	***x*** = [*g l*_1*r*_* l*_*a*_* l*_*b*_* w*_1_ *w*_2*r*_* w*_3*r*_* w*_4*r*_* w*_*a*_* w*_*b*_]^*T*^	***x*** = [*L*_*s*_* W*_*s*_* l*_3*r*_* w*_1_ *w*_2_ *w*_3_ *w*_4_ *w*_5_ *w*_*v*_]^*T*^
High-fidelity model^#^	~ 115,000 mesh cells; simulation time 350 s	~ 85,000 mesh cells; simulation time 90 s	~ 120,000 mesh cells; simulation time 150 s
Low-fidelity model^#^	~ 48,000 mesh cells; simulation time 125 s	~ 27,000 mesh cells; simulation time 33 s	~ 61,000 mesh cells; simulation time 68 s
Other parameters	*d*_1_ = *d* +|*w* − *w*_1_|, *d* = 1.0, *w*_0_ = 1.7, and *l*_0_ = 15	*L* = 2*dL* + *L*_*s*_, *L*_*s*_ = 4*w*_1_ + 4* g* + s + *l*_*a*_ + *l*_*b*_, *W* = 2*dL* + *W*_*s*_, *W*_*s*_ = 4*w*_1_ + 4* g* + *s* + 2*w*_*a*_, *l*_1_ = *l*_*b*_*l*_1*r*_, *w*_2_ = *w*_*a*_*w*_2*r*_, *w*_3_ = *w*_3*r*_*w*_*a*_, *w*_4_ = *w*_4*r*_*w*_*a*_	*d*_*L*_ = *d*_*W*_ = 10 mm, *L* = 2*d*_*L*_ + *L*_*s*_, *W* = 2*d*_*W*_ + 2*w*_1_ + (*W*_*s*_ − 2*w*_*f*_), *l*_1_ = *W*_*s*_/2, *l*_2_ = *l*_3_2^1/2^, *l*_3_ = *l*_3*r*_((*L*_*s*_* − w*_3_)/2 − *w*_4_/2^1/2^), *l*_*v*1_ = *l*_3_/3, *l*_*v*3_ = *L*_*s*_/2 − *w*_3_/2 − *l*_3_ + *l*_*v*1_; *w*_*f*_ = 1.15 mm
Operating bands	0.89–1.11 GHz	1.45–1.55 GHz	2.36–2.44 GHz 5.16–5.24 GHz
Maximum power split error	0.4 dB at 1 GHz	0.5 dB at 1.5 GHz	0.5 dB at 2.4 GHz 0.5 dB at 5.2 GHz
Nominal design	***x***^(0)^ = [4.50 11.08 21.81 0.65 0.94 0.86]^*T*^	***x***^(0)^ = [0.63 5.90 9.34 12.45 1.29 2.02 0.99 0.32 2.81 0.22]^*T*^	***x***^(0)^ = [25.05 0.85 0.76 1.90 1.23 0.36 0.71 0.30 0.30]^*T*^

^$^Parameters with subscript *r* are relative, and their deviations are recalculated accordingly in order to have the corresponding absolute parameters following the assumed probability distribution (here, Gaussian with variance of 0.017 mm).

^#^All EM models are implemented in CST Microwave Studio and evaluated using the time domain solver.

## Reference algorithms

The performance of the introduced optimization procedure is benchmarked against several methods that have been outlined in Table 2. All of these are surrogate-assisted techniques that represent different approaches to robust design using data-driven models. In Algorithm 1, EM model is entirely replaced by the kriging surrogate built in a sufficiently spacious domain, allocated in the vicinity of the nominal design. This approach is straightforward but the cost of constructing the model may be large owing to the extent of the domain. Algorithm 2 utilizes a sequential approximate optimization approach, with several local surrogates rendered along the optimization path, which allows for lowering the cost of individual model construction at the expense of repeating the process across a few iterations. Overall, this method is expected to offer computational savings over Algorithm 1, especially for parameter spaces of larger dimensions.

**Table 2:** Benchmark algorithms.

Algorithm	Description
1	Surrogate-assisted method using a metamodel established in a relatively large vicinity of the nominal design to enable sufficient relocation of the design during yield optimization The yield optimization task (8) is solved using a local optimization algorithm The metamodel is constructed using kriging^46^, in the interval domain [***x***^(0)^ − ***d***, ***x***^(0)^ + ***d***] The entries of the size vector ***d*** = [*d*_1_ … *d*_*n*_]^*T*^ are set to *d*_*k*_ = 10*d*_max_, *k* = 1, …, *n* (*d*_max_ is the maximum parameter deviation) Remarks: The above domain size is normally sufficient to conclude yield maximization in a single iteration. The method is simple to implement. The drawback is a potentially high cost of training data acquisition, especially for higher-dimensional parameter spaces
2	Procedure based on sequential approximate optimization (SAO) approach^35^. The problem (8) is solved iteratively as ${\mathbf{x}}^{(i + 1)} = \arg \mathop {\min }\limits_{{\mathbf{x}}} \{ - Y_{s}^{(i)} ({\mathbf{x}})\} \quad (17)$ where ***x***^(*i*)^, *i* = 0, 1, …, are approximations to the optimum design x* The yield *Y*_*s*_^(*i*)^ in the *i*th iteration, is estimated using a surrogate model established in the local domain [***x***^(*i*)^ − ***d***_*l*_, ***x***^(*i*)^ − ***d***_*l*_], ***x***^(*i*)^ = [*x*_1_^(*i*)^ … *x*_*n*_^(*i*)^]^*T*^, centred at the current iteration point The surrogates are constructed along the optimization path; thus, their domains can be smaller than for Algorithm 1; here, ***d***_*l*_ = [*d*_*l.*1_ … *d*_*l.n*_]^*T*^ is set to *d*_*l.k*_ = 3*d*_max_, *k* = 1, …, *n* Problem (17) is constrained to satisfy *x*_*k*_^(*i*)^ − *d*_*l.k*_ + *d*_max_ ≤ *x*_*k*_ ≤ *x*_*k*_^(*i*)^ + *d*_*l.k*_ − *d*_max_, *k* = 1, …, *n*, which allows to run Monte Carlo simulation within the region of validity of the metamodels Remarks: The advantage of this procedure is a lower cost of setting up the surrogate as compared Algorithm 1. Yet, several iterations are required to approach the optimum solution
3	Procedure based on the performance-drive modelling concept^21^ The surrogate is constructed in the domain spanned by the most relevant directions that affect the likelihood of satisfying the design requirements in the most significant manner, cf. Fig. 6 These directions are found through auxiliary local optimizations^21^ Remarks: The advantage of this method is low volume of the surrogate model domain, which is of sufficient size wherever necessary. Thus, Algorithm 3 effectively combines the advantages of Algorithms 1 and 2
4	Feature-based procedure embedded in the trust-region framework^38^ The overall optimization procedure is similar to the algorithm described in “Response features for low-cost yield estimation”, “Yield optimization using variable-resolution feature-based surrogates” and “Complete algorithm” sections; however, it is entirely based on high-fidelity EM model

**Figure 6:**
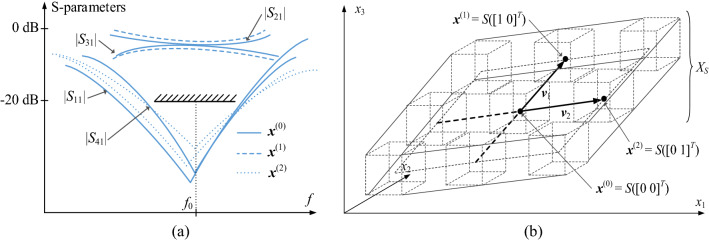
Performance-driven yield enhancement^21^: (**a**) *S*-parameters of a microwave coupler for design ***x***^(0)^ (nominal), design ***x***^(1)^ (degraded power split), and design ***x***^(2)^ (enhanced bandwidth at − 20 dB); for clarity, only relevant characteristics are shown; the directions important for yield manipulation are determined by designs ***x***^(1)^ and ***x***^(2)^; (**b**) surface *S*(***t***) parameterized by vector ***t*** = [*t*_1_ *t*_2_]^*T*^ is delimited by the designs ***x***^(0)^, ***x***^(1)^, and ***x***^(2)^; the model domain *X*_*S*_ is a union of intervals *S*_*I*_(***t***) for − 1 ≤ *t*_1_, *t*_2_ ≤ 1.

Algorithm 3 employs the performance-driven modeling concept^47^, in which the extent of the metamodel domain is larger along the important directions (here, representing more consequential variations of the circuit yield), and smaller along the remaining directions (see Fig. 6). This allows to combine the advantages of Algorithms 1 and 2, i.e., to conclude the robust design process using a single model, while maintaining relatively low cost of data acquisition. Finally, Algorithm 4 is the framework reported in^38^, which utilizes the same mechanisms as described in “Response features for low-cost yield estimation”, “Yield optimization using variable-resolution feature-based surrogates” and “Complete algorithm” sections, but the entire optimization process is conducted using the high-resolution EM model.

The purpose of the verification experiments is to analyze the performance indicators of the presented and the benchmark algorithms, in particular, the computational complexity and reliability. The latter is validated through EM-based Monte Carlo (MC) simulation, carried out with the use of 500 random points featuring the assumed probability distribution for input tolerances. While the number of points is restricted because of high cost of massive EM analyses, a significant consequence is that this leads to a decreased accuracy of MC, which is about two percent.

### Results and discussion

Table 3 gathers the yield-enhanced solutions for Circuits I through III found by the proposed variable-resolution feature-based procedure. Tables 4, 5, and 6 gather the comparison data for all the circuits and all benchmark algorithms. Observe that—as expected—the incorporation of variable-resolution models leads to further improvement of the cost efficacy of the yield optimization routine. It is already remarkably low for Algorithms 3 and 4, with the average number of high-fidelity EM simulations being 105 and 31, respectively. Yet, the proposed approach brings these numbers even lower, to the average of twenty, which corresponds to 36-percent saving over Algorithm 4, and 81-percent savings over Algorithm 3. As explained in “Yield optimization of microwave passives using multi-fidelity response features” section, this is due to the fact that most of EM analyses is executed to estimate the Jacobian matrix, and carrying out this task using the low-fidelity models reduces this cost by a factor of about three for the considered coupler circuits.

**Table 3:** Yield-optimized designs for Circuits I through III obtained using the proposed approach.

Circuit	Parameter vector
I	x* = [4.70 11.38 21.66 0.75 0.94 0.86]^*T*^
II	x* = [0.63 5.98 9.21 12.53 1.29 2.08 0.99 0.31 2.82 0.28]^*T*^
III	x* = [25.05 0.85 0.76 1.90 1.23 0.37 0.71 0.30 0.30]^*T*^

**Table 4:** Design centering results for Circuit I (Fig. 5a).

Optimization algorithm	Initial yield		Optimized yield		CPU cost^$^
	Estimated by surrogate model (%)	EM-based (%)	Estimated by surrogate model (%)	EM-based (%)	
Reference algorithm 1	50	42	100	97	400
Reference algorithm 2	45	42	97	97	200^#^
Reference algorithm 3	44	42	98	98	82
Reference algorithm 4	45	42	99	98	25
This work (“Yield optimization of microwave passives using multi-fidelity response features” section)	48	42	99	99	16.0^#^

^$^Optimization cost in number of EM simulations of the considered circuit.

^#^Equivalent number of high-fidelity EM simulations (actual number of analyzes was 6 high-fidelity and 28 low-fidelity).

**Table 5:** Design centering results for Circuit II (Fig. 5(b)).

Optimization algorithm	Initial yield		Optimized yield		CPU cost^$^
	Estimated by surrogate model (%)	EM-based (%)	Estimated by surrogate model (%)	EM-based (%)	
Reference algorithm 1	82	77	93	88	800
Reference algorithm 2	76	77	94	93	320
Reference algorithm 3	79	77	92	93	112
Reference algorithm 4	79	77	90	92	37
This work (“Yield optimization of microwave passives using multi-fidelity response features” section)	79	77	94	92	23.1^#^

^$^Optimization cost in number of EM simulations of the considered circuit.

^#^Equivalent number of high-fidelity EM simulations (actual number of analyzes was 7 high-fidelity and 44 low-fidelity).

**Table 6:** Design centering results for Circuit III (Fig. 5c).

Optimization algorithm	Initial yield		Optimized yield		CPU cost^$^
	Estimated by surrogate model (%)	EM-based (%)	Estimated by surrogate model (%)	EM-based (%)	
Reference algorithm 1	80	71	99	93	800
Reference algorithm 2	88	71	96	91	500
Reference algorithm 3	74	71	94	92	123
Reference algorithm 4	71	71	93	89	32
This work (“Yield optimization of microwave passives using multi-fidelity response features” section)	71	71	90	89	19.6^#^

^$^Optimization cost in number of EM simulations of the considered circuit.

^#^Equivalent number of high-fidelity EM simulations (actual number of analyzes was 6 high-fidelity and 30 low-fidelity).

In terms of design quality, the solutions obtained using the proposed approach are comparable to those identified using the benchmark methods. The same can be said about reliability, as confirmed through EM-based Monte Carlo simulations. It should be reiterated that the variance of the MC-estimated yield is relatively high (up to two percent), as emphasized before, due to a relatively low number of observables used in the process. This means that the yield differences of up to two or three percent are statistically insignificant.

**Figure 7:**
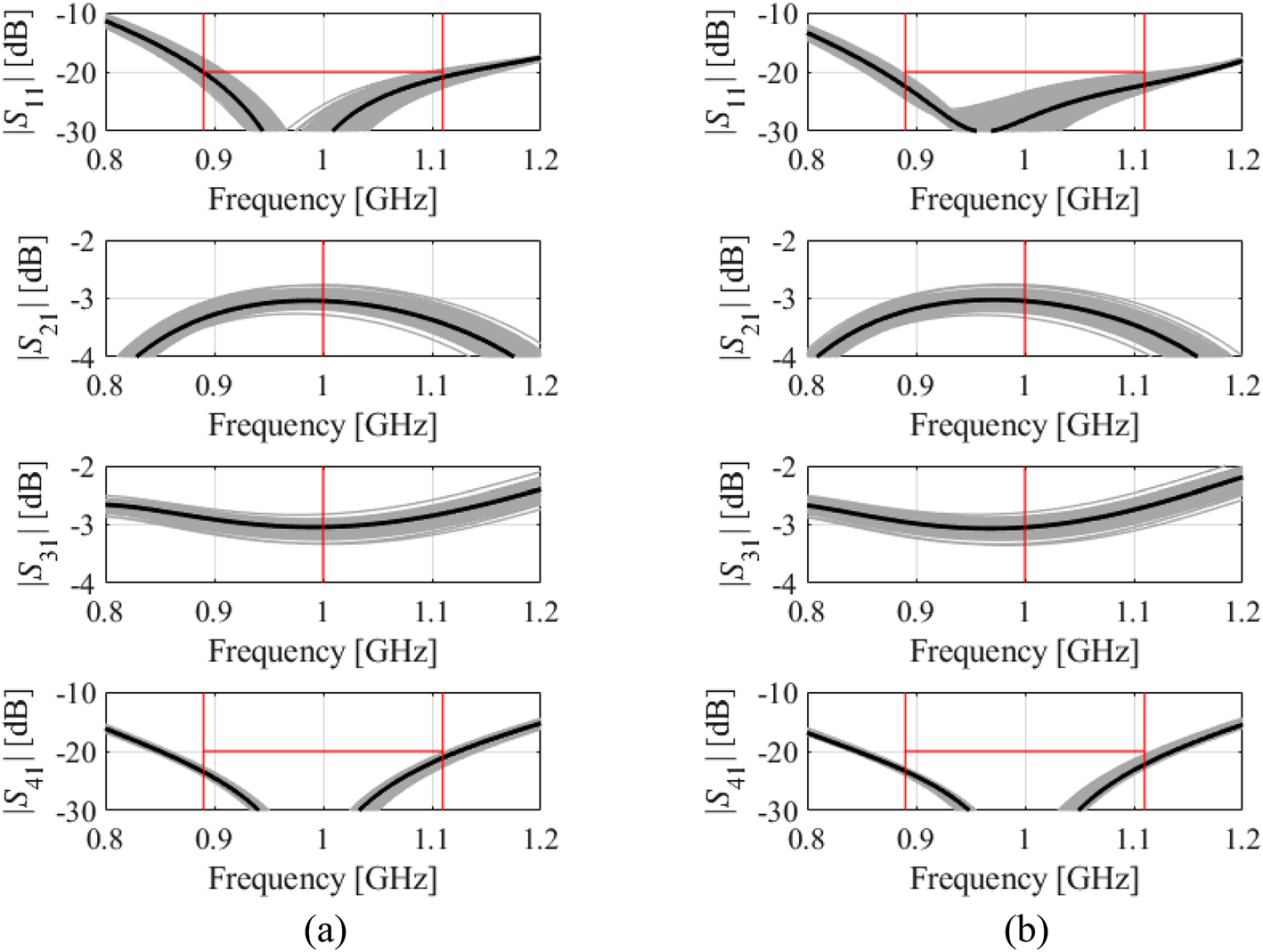
Visualization of EM-based Monte Carlo analysis for Circuit I: (**a**) at the nominal design, and (**b**) at the optimal design rendered with the use of the procedure introduced in this work. MC is executed using 500 random data points. Gray curves represent EM simulations, whereas the circuit characteristics at the nominal (**a**) and optimal design (**b**) are shown black.

**Figure 8:**
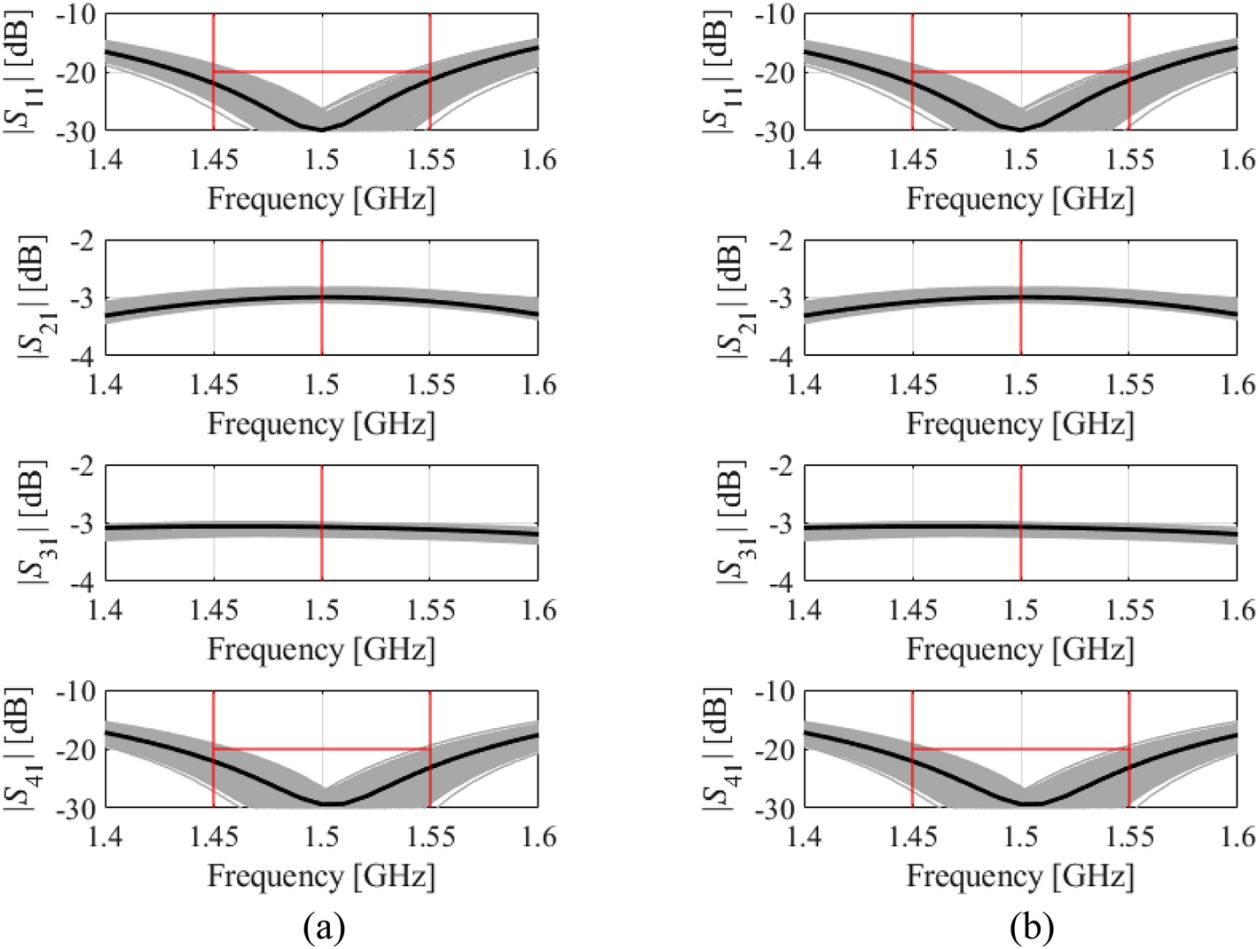
Visualization of EM-based Monte Carlo analysis for Circuit II (**a**) at the nominal design, and (**b**) at the optimal design rendered with the use of the procedure introduced in this work. MC is executed using 500 random data points. Gray curves represent EM simulations, whereas the circuit characteristics at the nominal (**a**) and optimal design (**b**) are shown black.

**Figure 9:**
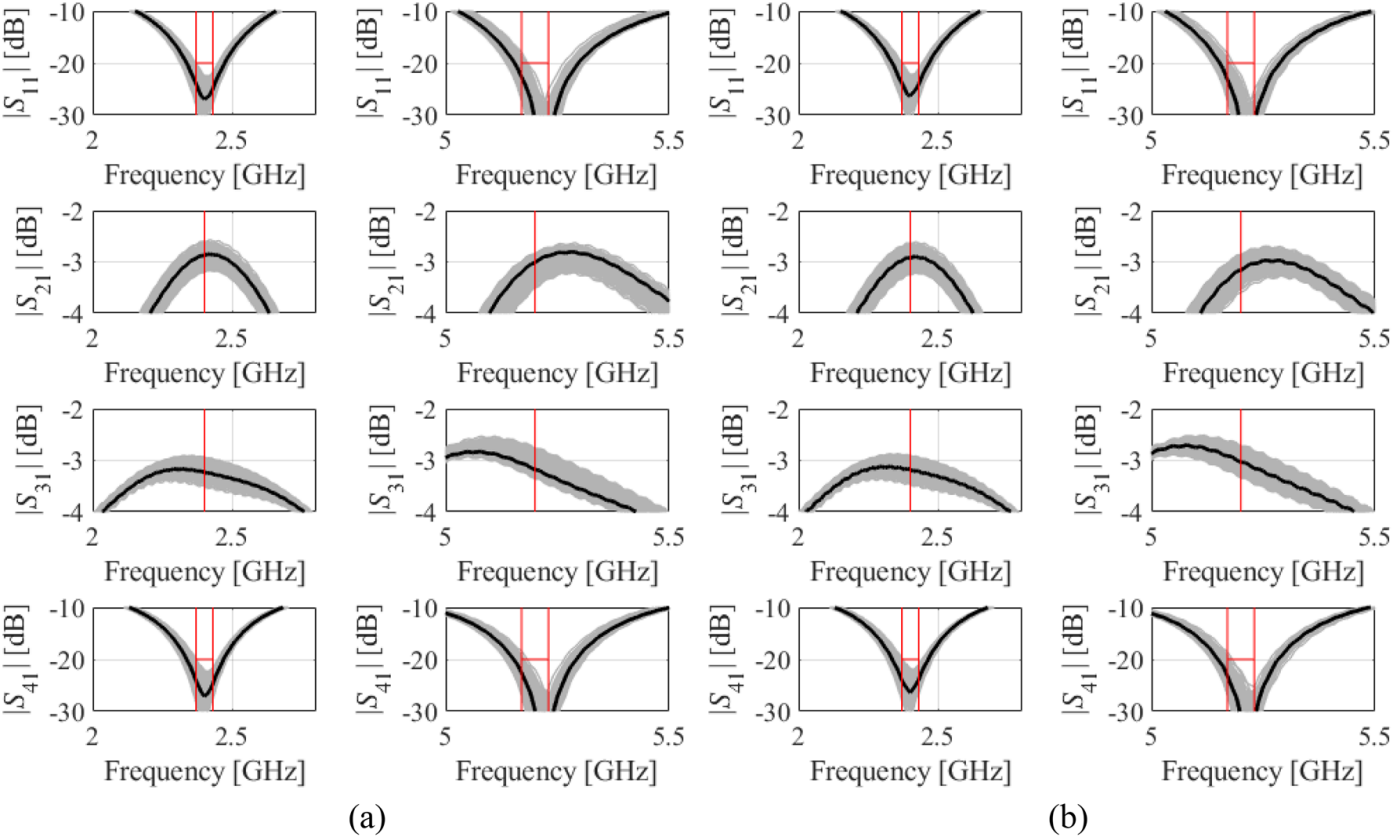
Visualization of EM-based Monte Carlo analysis for Circuit III: (**a**) at the nominal design, and (**b**) at the optimal design rendered with the use of the procedure introduced in this work. MC is executed using 500 random data points. Gray curves represent EM simulations, whereas the circuit characteristics at the nominal (**a**) and optimal design (**b**) are shown black.

It can also be noticed that the differences between MC- and surrogate-model-estimated yield values are the highest for Circuit III, which is because this circuit is the most difficult to model. For example, the relative RMS error of the surrogate used by Algorithm 1 is better than four percent for Circuits I and II, but it exceeds six percent for Circuit III, despite of using as many as 800 training data samples. Figures 7, 8 and 9 provide visualization of EM-based Monte Carlo simulation at the nominal and robust designs obtained using the proposed algorithm, for Circuit I, II, and III, respectively. Again, MC is based on 500 random samples.

Observe that recognition of the response features may prove problematic due to misshaped circuit responses, which may occur for design cases with large statistical variations. Nevertheless, as indicated by the results of EM-driven Monte Carlo simulations (Figs. 7, 8, 9), such a situation would might happen for the parameter variations of at least an order of magnitude larger than those assumed in the paper under review (i.e., *d*_max_ = 0.05 mm). Practically, for the PCB technology, such large variations are unrealistic, as this would mean, e.g., error of etching the circuit slits of around one millimeter (i.e., comparable to the slit width). The actual manufacturing procedures (chemical etching or mechanical milling) are considerably more accurate with the deviations corresponding to the levels assumed in this work.

**Figure 10:**
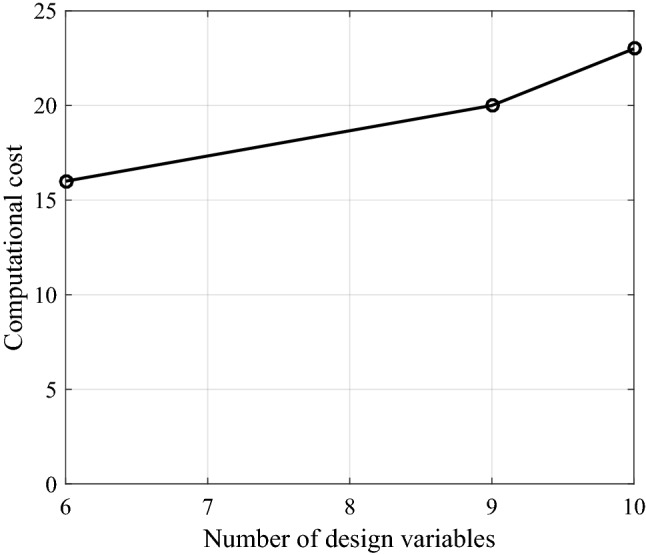
Computational cost of the yield optimization process using the proposed algorithm versus number of design variables for Circuit I, III, and II described by 6, 9, and 10 design variables.

The computational cost of our procedure amounts to around 16, 20 and 23 EM simulations for the structures featuring 6, 9, and 10 parameters (see Tables 4, 5, 6). Thus, the dependence of the cost on the number of design variables is close-to-linear: the ratio between the computational cost for Circuit II (described by the largest number of design variables) and Circuit I (described by the smallest number of design variables) equals around 1.5 and it is almost equal to the ratio between the respective numbers of design variables. This suggests that for higher-dimensionality cases, the computational cost of the proposed procedure would be increased proportionally to the number of geometry parameters describing the microwave component of interest. The relationship between the computational cost and the number of design variables is visualized in Fig. 10.

The remarkable cost reduction obtained using our approach is achieved at the expense of limiting the scope of its applicability to structures whose responses feature discernible characteristics points, which should be defined to enable estimation of the yield. The examples of such structures include microwave filters or impedance matching transformers. In the case of microwave filters, the response features may be defined as the local maxima of the return loss within the pass-band, as well as the crossing points at the edge of the pass-band. Similar definition may be employed in the case of impedance matching transformers, where possible definition of the response features includes local maxima of the reflection characteristics, as well as points defining the bandwidth at the assumed target level, e.g. − 20-dB. Overall, the proposed methodology may be not as versatile as other frameworks that do not impose any restraints on the response structure of the component under design. Yet, the characteristics of many real-world microwave passives are inherently structured. Consequently, the employment of the feature-based techniques such as the proposed one is only slightly hindered by the aforementioned factors.

## Conclusion

This work introduced, a novel technique for cost-efficient optimization of the fabrication yield of microwave passives. The presented methodology employs an ensemble of acceleration mechanisms, including regression-based surrogate modeling at the level of response features, as well as variable-fidelity EM simulations. Both permit reliable and fast estimation of the yield, maximization of which exploits a sequential approximate optimization paradigm, and also the trust-region framework to govern design relocation and secure convergence of the procedure. Numerical verification of our procedure has been realized with the use of three microstrip couplers. Its efficacy has been compared to several surrogate-assisted algorithms. The results demonstrate that incorporation of the aforementioned algorithmic tools gives a competitive edge over the benchmark, with computational savings as high as over ninety percent. In absolute terms, the average cost of yield optimization corresponds to only twenty EM circuit simulations at the high-fidelity level, which is 36 percent cheaper than for the feature-based algorithm exclusively using high-fidelity models (being one of the benchmark methods). At the same time, the reported speedup does not compromise the yield evaluation reliability, as corroborated using EM-based MC analysis. The proposed framework is simple to implement, and can be viewed as a CPU-efficient replacement of conventional statistical design methods, particularly for circuits whose responses exhibit easily distinguishable characteristic points.

## Data Availability

The datasets generated during and/or analysed during the current study are available from the corresponding author on reasonable request.
